# Mapping Systematic Reviews on Atopic Eczema—An Essential Resource for Dermatology Professionals and Researchers

**DOI:** 10.1371/journal.pone.0058484

**Published:** 2013-03-11

**Authors:** Masaki Futamura, Kim S. Thomas, Douglas J. C. Grindlay, Elizabeth J. Doney, Donna Torley, Hywel C. Williams

**Affiliations:** 1 Centre of Evidence Based Dermatology, University of Nottingham, King’s Meadow Campus, Nottingham, United Kingdom; 2 Division of Allergy, National Center for Child Health and Development, Tokyo Japan; 3 Centre for Evidence-based Veterinary Medicine, University of Nottingham, Sutton Bonington Campus, Leicestershire, United Kingdom; 4 Alan Lyell Centre for Dermatology, Southern General Hospital Glasgow, Glasgow, United Kingdom; CNRS-University of Toulouse, France

## Abstract

**Background:**

Many research studies have been published on atopic eczema and these are often summarised in systematic reviews (SRs). Identifying SRs can be time-consuming for health professionals, and researchers. In order to facilitate the identification of important research, we have compiled an on-line resource that includes all relevant eczema reviews published since 2000.

**Methods:**

SRs were searched for in MEDLINE (Ovid), EMBASE (Ovid), PubMed, the Cochrane Database of Systematic Reviews, DARE and NHS Evidence. Selected SRs were assessed against the pre-defined eligibility criteria and relevant articles were grouped by treatment category for the included interventions. All identified systematic reviews are included in the Global Resource of EczemA Trials (GREAT) database (www.greatdatabase.org.uk) and key clinical messages are summarised here.

**Results:**

A total of 128 SRs reviews were identified, including three clinical guidelines. Of these, 46 (36%) were found in the Cochrane Library. No single database contained all of the SRs found. The number of SRs published per year has increased substantially over the last thirteen years, and reviews were published in a variety of clinical journals. Of the 128 SRs, 1 (1%) was on mechanism, 37 (29%) were on epidemiology, 40 (31%) were on eczema prevention, 29 (23%) were on topical treatments, 31 (24%) were on systemic treatments, and 24 (19%) were on other treatments. All SRs included searches of MEDLINE in their search methods. One hundred six SRs (83%) searched more than one electronic database. There were no language restrictions reported in the search methods of 52 of the SRs (41%).

**Conclusions:**

This mapping of atopic eczema reviews is a valuable resource. It will help healthcare practitioners, guideline writers, information specialists, and researchers to quickly identify relevant up-to-date evidence in the field for improving patient care.

## Background

Atopic eczema (AE), also known as atopic dermatitis, is a common disease that attracts considerable research interest [1], [2]. Data from published epidemiological research and clinical trials, including randomized controlled trials (RCTs), are exponentially increasing [3]. RCTs are the recognised gold standard for assessing the effectiveness of interventions [4], and these have recently been collated into an openly accessible on-line database of eczema trials, the Global Resource of EczemA Trials (GREAT) database (www.greatdatabase.org.uk) [5].

However, relying on single RCTs is hazardous [6], and systematic reviews (SRs) that collate information from individual studies to provide a more reliable form of evidence are an essential tool for healthcare practitioners.

The aim of this project was to identify and provide easy access to all atopic eczema systematic reviews in a convenient “one-stop shop” in order to facilitate the practice of evidence-based dermatology amongst healthcare practitioners, guideline writers, information specialists, and researchers. In ensuring the easy identification of all published SRs, we hope to reduce unnecessary duplication of effort, and to assist with the identification of potential areas that require an up-to-date review.

As part of the process to build a SR resource, we have been regularly reviewing all SRs on AE published since 2000 and these have been summarised as Annual Evidence Updates published each year [7]–[11]. A similar resource summarising SRs on acne vulgaris has been produced and maintained since 2007, and is available from the website for the Centre of Evidence Based Dermatology, University of Nottingham (www.nottingham.ac.uk/dermatology). This mapping of SRs has been used to produce clinical evidence updates in acne [12]–[14], and similar updates have been published for psoriasis [15], [16] and skin cancer [17], [18].

This paper provides an opportunity to direct healthcare practitioners and researchers to the relevant SRs on AE for different topic areas, and to highlight some of the key messages to have emerged from the last 13 years of AE research.

## Methods

### Search Dates

Searches were conducted for studies published from 1^st^ January 2000 to 31^st^ December 2012. The last search was conducted on 16^th^ January 2013 in order to allow for the inclusion of studies published in 2012, although it is possible that some reviews published towards the end of 2012 may have been omitted if they have not been indexed yet.

### Sources Searched

The following electronic databases were searched: MEDLINE (Ovid), EMBASE (Ovid), PubMed, Cochrane Database of Systematic Reviews, Database of Abstracts of Reviews of Effects (DARE) and NHS Evidence. Searches were conducted by a trained information specialist or librarian (DJCG or EJD).

### Search Terms

The following search terms were used in all the databases: “eczema”, “atopic dermatitis” and “neurodermatitis”. The SIGN (Scottish Intercollegiate Guidelines Network) SR filters were used to identify SRs in MEDLINE and EMBASE (see Appendix S1 & S2 in Appendixes S1). The PubMed Clinical Queries SR filter was used for the PubMed search. There were no language restrictions in our searches.

### Identification of SRs

An SR was defined from the Glossary of Terms in the Cochrane Collaboration as “A review of a clearly formulated question that uses systematic and explicit methods to identify, select, and critically appraise relevant research, and to collect and analyse data from the studies that are included in the review. Statistical methods (meta-analysis) may or may not be used to analyse and summarise the results of the included studies” [19].

All citations from the searches were handsearched by a single investigator (MF or DJCG) by reading titles and abstracts to identify SRs, and potential SRs, relevant to AE. The following exclusion criteria were used: 1) not relevant for the clinical topic, 2) non-review article, 3) conference abstract only, or 4) methodology unclear preventing potential replication or validation of results. Reviews that did not name the databases that had been searched or the dates of the search were also excluded. In order to be inclusive, we did not exclude SRs because they had 1) searched a single database, 2) used a single data extractor, 3) applied language restrictions, or 4) were not pre-registered. Clinical guidelines were included if a SR had been carried out and published as part of the production process. The final decision on whether publications were to be included as a SR was made by HCW.

### Mapping of Reviews

The identified SRs were sorted into six categories: mechanism, epidemiology, prevention, topical treatments, systemic treatments, and other treatments. Updated reviews were counted as a single SR if they were published in the same journal as the earlier version, and for these the latest publication date was used [20]. In the tables, citations (with links to on-line records) are given by category and topic area, and ordered according to the date of the last search. Where a SR covered more than one topic area, it was listed under all relevant topics. Details of databases used for the SR and any language restrictions applied are also listed. The country of origin for all SRs was defined as the location of the institution for the first author.

### Current AE Guidelines

Current guidelines on AE were checked to confirm how many of the identified SRs had been used to inform relevant clinical guidelines. We searched for AE guidelines published since 2007 from the National Guidelines Clearing House website (http://guideline.gov/) in addition to PubMed. We selected guidelines which 1) were written in English or had references mainly written in English, 2) were published in medical journals or on a freely accessible website, and 3) were written on behalf of governmental or national organizations. For each identified guideline, the reference list was scrutinised in order to identify the number of relevant SRs that had been cited.

### Value of the SR Mapping

In order to assess the potential value of this mapping of SRs in the dermatology community, we conducted an on-line survey between 19^th^ November and 4^th^ December 2012. This anonymous survey was open for two weeks and was performed on-line. Approach letters were sent to 480 members of the UK Dermatology Clinical Trial Network (UK DCTN) (http://www.ukdctn.org/) and to 140 members of the international Harmonizing Outcome Measures for Eczema (HOME) initiative (http://www.homeforeczema.org/). Participants were asked to comment on the potential uses of the map of SRs and to assess how useful the resource would be in their own work.

## Results

### Overall Characteristics

**Figure 1:**
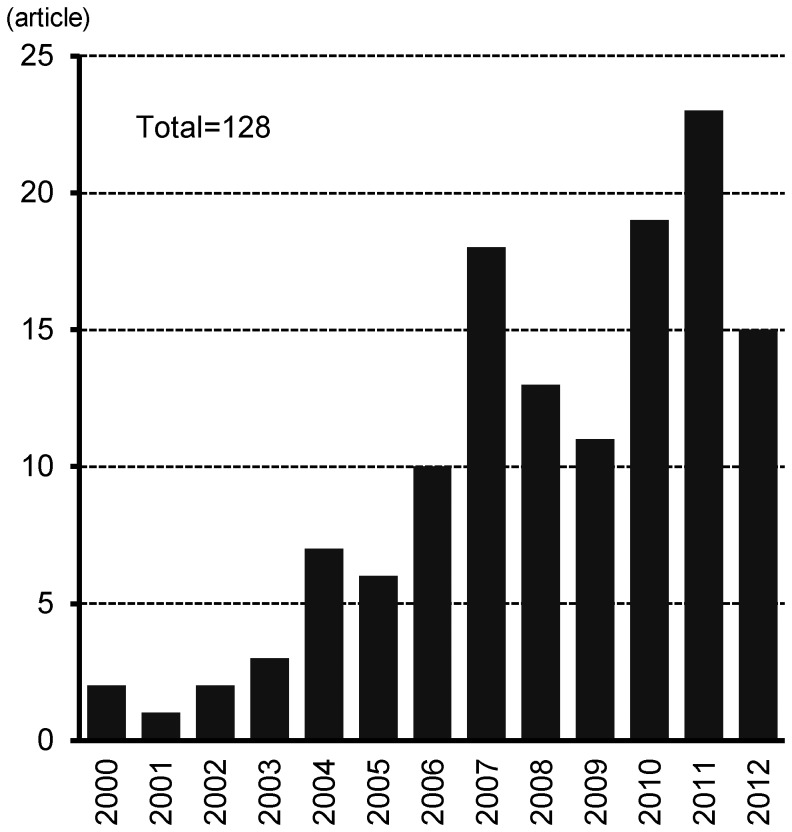
Numbers of published systematic reviews by year.

Our search identified 128 SRs on AE published between 2000 and 2012 (see Figure 1). None of the databases searched contained all of the 128 included SRs. Forty six (36%) were found in the Cochrane Library, 102 (80%) were found in MEDLINE, 113 (88%) were found in EMBASE, 107 (84%) from PubMed, and 53 (41%) from NHS Evidence.

Three non-English SRs (one German and four Chinese) were included. Three clinical guidelines were included as they fulfilled our inclusion criteria of being guidelines containing their own systematic reviews [21]–[23].

**Figure 2:**
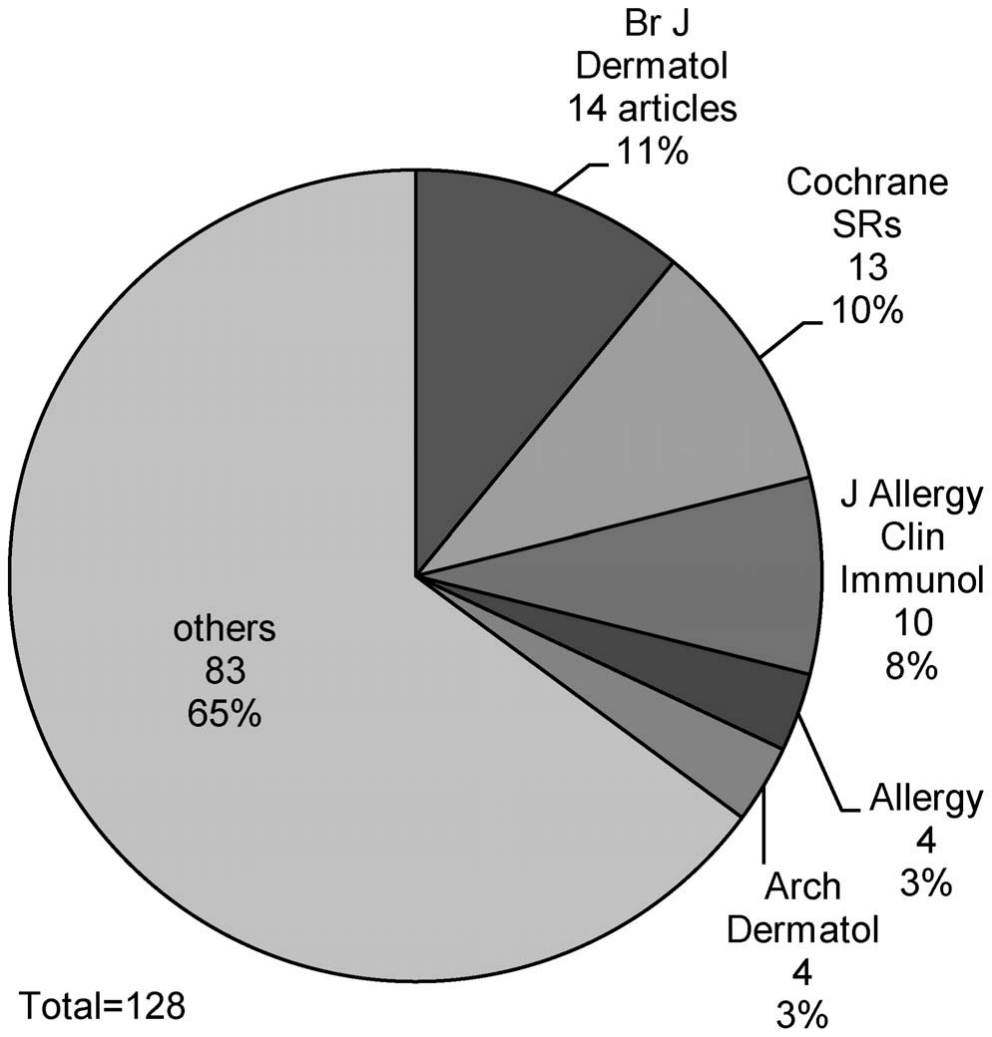
Journals of publication.

**Figure 3:**
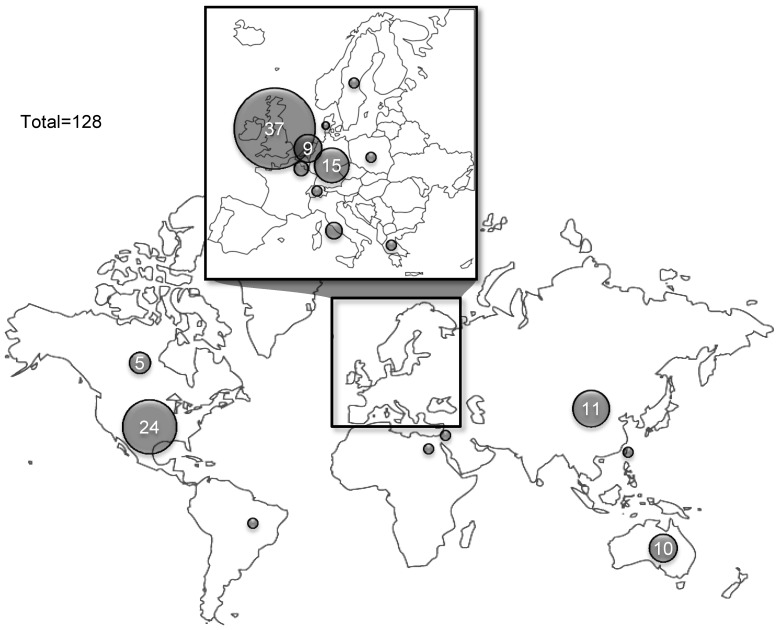
Geographic distribution of author’s institutions.

Eighty one SRs were published in the last five years alone – equating to an average of 16 SRs per year. The most common places for SRs to be published were the *British Journal of Dermatology* (14) and the *Cochrane Database of Systematic Reviews* (13) (see Figure 2). However, SRs were published in many journals, and the five most commonly used journals accounted for less than 40% of the total number of reviews. Thirty seven (29%) of the SRs originated in the United Kingdom, while 24 (19%) came from the United States, and 15 (12%) were from Germany (see Figure 3).

### Topic Areas of Systematic Reviews

The categories covered by the SRs were mechanism (1; 1%), epidemiology (37; 29%), prevention (40; 31%), topical treatments (29; 23%), systemic treatments (31; 24%), and other treatments (24; 19%). Each topic had between 1 and 24 relevant SRs (see Table 1). In recent years, there has been increasing interest in prevention (28% between 2000 and 2007, 33% between 2008 and 2012).

**Table 1:** Number of systematic reviews by category.

Category	Topic (articles)
Mechanism	autoreactivity (1)
Epidemiology	aetiology/risk factor (15), disease impact/evaluation (14), prevalence/co-morbidity (12)
Prevention	dietary/supplement (28), breastfeeding (15), maternal diet (12), other prevention (4)
Topical treatments	calcineurin inhibitor (21), corticosteroid (12), emollient (7), antimicrobial (6), occlusive therapy (4), other topical (5)
Systemic treatments	dietary/supplement (20), immunotherapy/desensitisation (10), immune modulator (7), antimicrobial (6), anti-histamine/anti-allergic (5), corticosteroid (3), other systemic (4)
Other treatments	Chinese herb (10), psychological/education (9), complementary/alternative (9), phototherapy (6), clothing (6), environmental control (5)

### Quality of SRs

The number of databases search in a SR can be one indicator of quality of the review. Most of the included SRs searched more than one database (106 SRs; 83%). The most commonly searched database was MEDLINE (used in all SRs), followed by EMBASE (used in 78 SRs; 61%). No language restrictions were reported in 52 SRs (41%).

A meta-analysis was conducted in 71 of the SRs (56%). Five of the thirteen Cochrane SRs had been updated at least once [24]–[26]. None of the other SRs had been updated.

### Summary of Clinical Implications from the Reviews

For the last five years, Annual Evidence Updates have been produced that summarise the recent evidence from published SRs [7]–[11]. The key clinical messages from these updates, and from other SRs published between August 2010 and December 2012 are briefly outlined in Table 2.

**Table 2:** Evidence from recent systematic reviews.

		Epidemiology		Prevention		Treatment
2006–2007 [8]	✓	around a third of children with eczema developed asthma by 6 yrs old	?	delayed introduction of solids	✓	educational support in a nurse-led clinic
	?	textiles, irritants and detergents in causing eczema flares	X	avoiding allergenic foods duringpregnancy	✓	psychological and educational intervention
			X	hydrolyzed formulae or soy formulae	✓	short term wet wraps for induction of remission in moderate/severe eczema
					✓	oral cyclosporine for induction of remission in severe eczema
					✓	UVA1 for acute eczema
					✓	narrowband UVB for chronic eczema
					?	IVIG and infliximab
					X	more than once daily application of TCS
					X	evening primrose oil
2007–2008 [9], [10]	✓	UK Working Party’s diagnostic criteria arethe most extensively validated	✓	probiotics in infants born to atopicparents	✓	tacrolimus more effective than weak TCS or pimecrolimus
	✓	sufficiently tested outcome measures are SCORAD, EASI and POEM	?	prebiotics	✓	pimecrolimus better than plain grease
	✓	association with adverse psychologicalfactors early in life	?	breastfeeding	✓	pimecrolimus less effective than potent TCS or tacrolimus
	✓	association with FLG mutation	X	keeping a furry pet early in life	?	subcutaneous desensitization
	✓	decrease the risk of developing a glioma			?	exclusion diets, few-food diets or elemental diets
	✓	leading family sleep loss, anxiety and depression			?	anti-staphylococcus intervention for eczema
	✓	direct cost can be large			X	probiotics
	X	association with caesarean section				
2008–2009 [11]	✓	association with FLG mutation	X	exclusive breastfeeding for more than 3 months	?	dietary restrictions of certain foods
	?	relationship with TGF level in breast milk	X	omega-3 and omega-6 oils	?	long term safety of tacrolimus
					X	probiotics
2009–2010 [12]	✓	inverse relation with glioma/ALL	✓	partially hydrolysed formulas	✓	tacrolimus, pimecrolimus for children
	✓	association with ADHD	?	organic foods	?	bath emollients
	✓	increase risk when living in urban	?	fish or fish-oil supplementation	?	tacrolimus in treating pruritus
	?	association with multiple sclerosis			?	dry and wet occlusion
					?	silk clothing
					?	anti-staphylococcus intervention for eczema
2010–2011*	✓	inverse relation with meningioma	✓	probiotics with lactic acid bacteria	✓	proactive treatment for flare prevention
	✓	increase risk with antibiotics use	?	prebiotics	✓	tacrolimus as effective as mild/moderate TCS
	✓	decrease risk when keeping dogs	X	omega-3 oils during pregnancy	✓	tacrolimus more effective than pimecrolimus
	?	association with antioxidant status			?	patient education
	?	increase risk with mould exposure			?	coal tar
	X	decrease risk with childhood vaccination			?	azathioprine, Efalizumab
					?	homeopathy, Chinese herb, botanical extracts
					?	house dust immunotherapy
2012**	✓	increasing the prevalence in Africa, eastern Asia, western Europe and parts of northern Europe between1990 and 2010	✓	probiotics during pregnancy	✓	calicineurin inhibitor for pruritus
	✓	role of autoreactivity in driving disease exacerbation (Mechanism)	?	probiotics only in infant	?	immunotherapy
	?	defining incident cases in prevention trials	?	vitamin D during pregnancy	?	omalizumab
			X	avoiding allergenic foods duringpregnancy	?	homeopathy
			X	exclusive breastfeeding for more than 3 months	X	dietary supplement (oils, zinc, vitamin)

ADHD, attention deficit hyperactivity disorder; ALL, acute lymphoblastic leukaemia; EASI, the Eczema Area and Severity Index; FLG, filaggrin; HDM, house dust mites; IVIG, intravenous immunoglobulin; POEM, the Patient Oriented Eczema Measure; SCORAD, SCORing Atopic Dermatitis; TCS, topical corticosteroid; TGF, transforming growth factor, * between August 2010 and December 2011, ** updated on 16^th^ January 2013.

✓; probably effective based on systematic review evidence.

?; not clear, or limited evidence to recommendation.

X; unlikely to be effective based on systematic review evidence.

### SRs in Guidelines

We reviewed 12 guideline references relevant to AE that had been published since 2007 (Table 3) [22], [23], [27]–[37]. The guidelines most likely to cite SRs were the SIGN guideline on the management of AE in primary care (22 SRs) and the NICE guideline for the management of children with AE (14 SRs). However, some guidelines cited very few or no SRs, the reasons for which are unclear.

**Table 3:** Systematic reviews in guidelines on atopic dermatitis published since 2007.

Developer (Country/area)	Year	Target, Topics	Total references	Systematic Reviews (%)
NICE (UK) [22]	2007	Children	550	14 (2.5)
AAP (USA) [27]	2008	Prevention	63	5 (7.9)
AAP (USA) [28]	2008	Children	112	4 (3.6)
DSSA (South Africa) [29]	2008	Adults	168	10 (6.0)
DDG (German) [30]	2009	–	280	6 (2.1)
JDA (Japan) [31]	2009	–	66	0 (0.0)
EADV (Europe)* [32]	2010	–	135	5 (3.7)
AAAAI (USA)^**^ [33]	2011	Immunotherapy	6^†^	1 (16.7)
JSA (Japan) [34]	2011	–	32	0 (0.0)
SIGN (UK) [23]	2011	Primary care	62	22 (35.5)
BAD (UK)^***^ [35]	2012	–	22	1 (4.5)
EDF (Europe)^****^ [36], [37]	2012	–	363	7 (1.9)

AAAAI, American Academy of Allergy, Asthma and Immunology; AAP, American Academy of Pediatrics; BAD, British Association of Dermatologists; DDG, Deutschen Dermatologischen Gesellschaft [German Society of Dermatology]; DSSA, the Dermatological Society of South Africa; EADV, European Academy of Dermatology and Venereology; EDF, European Dermatology Forum; JAD, Japanese Dermatological Association; JSA, Japanese Society of Allergology; NICE, National Institute for Health and Clinical Excellence; SIGN, Scottish Intercollegiate Guidelines Network.

* also on behalf of the European Task Force on Atopic Dermatitis (ETFAD), ^**^also on behalf of the American College of Allergy, Asthma & Immunology (ACAAI) and the Joint Council of Allergy, Asthma & Immunology (JCAAI), ^***^also on behalf of Royal College of General Practitioners (RCGP), ^****^also on behalf of EADV, ETFAD, European Federation of Allergy (EFA), European Society of Paediatric Dermatology (ESPD), and Global Allergy and Asthma European Network (GA2LEN), ^†^relevant articles to atopic eczema.

### Usefulness of the Resource

In response to an on-line survey of dermatology health care professionals, 123 participants responded (91 members of the UK DCTN and 32 non-UK DCTN members of the HOME initiative). Overall, 112 (91%) of responders felt that the ability to identify relevant SRs quickly would be useful for their work, and 110 (90%) rated the mapping of SRs as being either ‘very useful’ or ‘somewhat useful’. General comments in response to the survey included: “This would be an invaluable source and a great asset for dermatologists” and “This is also useful for patients who can understand English”.

### Availability

Full links and citations to reviews relating to the treatment of eczema are included in the GREAT Database. This freely accessible database includes all RCTs of AE interventions published since 1966, and the SRs published since 2000 that are the topic of this paper. A full list of the SRs can be downloaded as a PDF file (available at http://www.nottingham.ac.uk/dermatology).

## Discussion

### Main Findings

For this review, we focused on one of the most prevalent and commonly researched skin diseases, and found 128 SRs published in the last thirteen years. The number of SRs published in recent years has increased significantly – more than 70% had been published since 2008. Although our eligibility criteria were broad, many articles were excluded because of unclear methodology, especially those published more than five years ago. To address the sub-optimal reporting of SRs, quality of reporting standards have been developed and published such as QUOROM (QUality Of Reporting Of Meta-analyses) in 1999 [38], and PRISMA (Preferred Reporting Items for Systematic reviews and Meta-Analyses) in 2009 [39]. Adherence to these reporting guidelines may explain the apparent increase in the number of eligible SRs included in this review in recent years.

In common with SRs of other diseases [40], the majority of SRs focused on treatments. However, prevention of AE was an increasingly frequent topic in recent years, possibly reflecting a growing research interest in public health.

SRs on AE were found in many different journals, making it difficult for health care practitioners and researchers to identify them easily. Although the Cochrane Library is a reliable source for identifying high-quality SRs [41], [42], it did not contain all the relevant reviews. Similarly, MEDLINE, the most commonly searched bibliographic database [43], did not contain seven of our included reviews. It was time consuming to identify non-Cochrane SRs in the bibliographic databases, and distinguishing true systematic reviews from other clinical reviews was sometimes difficult. From this we conclude that, as when searching for clinical trials to include in a SR, multiple databases should be used to find all relevant SRs on a given topic.

It would seem that many clinical guidelines are produced without reference to relevant and up-to-date SRs. Although it is not possible to establish why this might be, it is clearly important to promote the availability of this mapping of reviews as a resource for future guideline writers in order to ensure that clinical practice is based on the best available evidence.

### Strengths and Limitations

We searched six bibliographic databases. SRs were only included in this review if they fulfilled our eligibility criteria. Whilst we might have missed some SRs with non-English abstracts, we believe this review to be the most comprehensive summary of important SRs on AE in the world [44].

The importance of being able to identify relevant SRs quickly was supported by the dermatology professionals and researchers who responded to our survey, and so this resource will be maintained on an annual basis.

### Conclusions

This paper describes a collection of SRs on AE which provide a unique resource that will substantially reduce the amount of time and effort spent in searching for high-quality information by healthcare practitioners, guideline writers, information specialists, and researchers. This paper also summarises the key clinical messages to have emerged from these reviews over the last decade. The resource will be continually updated in the future (available through the Centre of Evidence Based Dermatology’s website and the GREAT Database), ensuring that the information remains up-to-date and relevant to the needs of the clinical community.

## Supporting Information

Appendixes S1 **Appendix S1. Search strategies of Ovid MEDLINE. Appendix S2. Search strategies of Ovid Embase.**
(DOCX)

Checklist S1 **PRISMA checklist.**
(DOCX)
