# Supplementary material for: Mapping Systematic Reviews on Atopic Eczema—An Essential Resource for Dermatology Professionals and Researchers
Source: PLoS One. 2013 Mar 11;8(3):e58484. doi: 10.1371/journal.pone.0058484 (PMC3594299; doi:10.1371/journal.pone.0058484)
Supplement: Appendixes S1 — Appendix S1. Search strategies of Ovid MEDLINE. Appendix S2. Search strategies of Ovid Embase. (DOCX) [file pone.0058484.s001.docx]

### Appendix 1 – Search strategies of Ovid MEDLINE

1. meta analy$.tw.

2. metaanaly$.tw.

3. (systematic adj (review$1 or overview$1)).tw.

4. exp "Review Literature as Topic"/

5. Meta-Analysis as Topic/

6. Meta-Analysis/

7. or/1-6

8. cochrane.ab.

9. embase.ab.

10. (psychlit or psyclit).ab.

11. (psychinfo or psycinfo).ab.

12. (cinhal or cinahl).ab.

13. science citation index.ab.

14. bids.ab.

15. cancerlit.ab.

16. or/8-15

17. reference list$.ab.

18. bibliograph$.ab.

19. hand-search$.ab.

20. relevant journals.ab.

21. manual search$.ab.

22. or/17-21

23. selection criteria.ab.

24. data extraction.ab.

25. 23 or 24

26. "Review"/

27. 25 and 26

28. Comment/

29. Letter/

30. Editorial/

31. Animals/

32. Humans/

33. 31 not (31 and 32)

34. or/28-30,33

35. 7 or 16 or 22 or 27

36. 35 not 34

37. atopic.mp.

38. dermatitis.mp.

39. 37 and 38

40. eczema.mp.

41. neurodermatitis.mp.

42. or/39-41

43. 36 and 42

### Appendix 2 – Search strategies of Ovid Embase

1. exp meta analysis/

2. ((meta adj analy$) or metaanalys$).tw.

3. (systematic adj (review$1 or overview$1)).tw.

4. or/1-3

5. cancerlit.ab.

6. cochrane.ab.

7. embase.ab.

8. (psychlit or psyclit).ab.

9. (psychinfo or psycinfo).ab.

10. (cinahl or cinhal).ab.

11. science citation index.ab.

12. bids.ab.

13. or/5-12

14. reference lists.ab.

15. bibliograph$.ab.

16. hand-search$.ab.

17. manual search$.ab.

18. relevant journals.ab.

19. or/14-18

20. data extraction.ab.

21. selection criteria.ab.

22. 20 or 21

23. review.pt.

24. 22 and 23

25. letter.pt.

26. editorial.pt.

27. animal/

28. human/

29. 27 not (27 and 28)

30. or/25-26,29

31. 4 or 13 or 19 or 24

32. 31 not 30

33. atopic.mp.

34. dermatitis.mp.

35. 33 and 34

36. eczema.mp.

37. neurodermatitis.mp.

38. or/35-37

39. 32 and 38
